# Supplementary material for: Coverage and system efficiencies of insecticide-treated nets in Africa from 2000 to 2017
Source: eLife. 2015 Dec 29;4:e09672. doi: 10.7554/eLife.09672 (PMC4758960; doi:10.7554/eLife.09672)
Supplement: Supplementary file 1. — HH = households; CAR = Central African Republic; DRC = Democratic Republic of Congo; STP = São Tomé and Príncipe. DOI: http://dx.doi.org/10.7554/eLife.09672.011 [file elife-09672-supp1.docx]

| **Country** | **Year** | **Type** | **HH size** | **No. ITNs owned** | **Type of ITN: conventional** | **Type of ITN: LLINs** | **Net type:** | **Individuals' usage of ITNs** | **Source** | **Ref** |
| --- | --- | --- | --- | --- | --- | --- | --- | --- | --- | --- |
|  |  |  |  |  |  |  | **all or one?** |  |  |  |
| Angola | 2006/07 | MIS | Yes | Yes | Yes | Yes | All | Yes | <http://measuredhs.com> | [1] |
| Angola | 2011 | MIS | Yes | Yes | Yes | Yes | All | Yes | <http://measuredhs.com> | [2] |
| Benin | 2006 | DHS | Yes | Yes | Yes | No | All | Yes | <http://measuredhs.com> | [3] |
| Benin | 2011/12 | DHS | Yes | Yes | Yes | Yes | All | Yes | <http://measuredhs.com> | [4] |
| Burkina Faso | 2003 | DHS | Yes | Yes | Yes | Yes | All | Yes | <http://measuredhs.com> | [5] |
| Burkina Faso | 2006 | MICS | Yes | No | Yes | Yes | One | No | <http://www.childinfo.org> | [6] |
| Burkina Faso | 2010 | DHS | Yes | Yes | Yes | Yes | All | Yes | <http://measuredhs.com> | [7] |
| Burundi | 2005 | MICS | Yes | No | Yes | Yes | One | No | <http://www.childinfo.org> | [8] |
| Burundi | 2010 | DHS | Yes | Yes | Yes | Yes | All | Yes | <http://measuredhs.com> | [9] |
| Burundi | 2012 | MIS | Yes | Yes | Yes | Yes | All | Yes | <http://measuredhs.com> | [10] |
| Cameroon | 2004 | DHS | Yes | Yes | Yes | Yes | All | Yes | <http://measuredhs.com> | [11] |
| Cameroon | 2011 | DHS | Yes | Yes | Yes | Yes | All | Yes | <http://measuredhs.com> | [12] |
| Congo | 2005 | DHS | Yes | Yes | Yes | Yes | All | Yes | <http://measuredhs.com> | [13] |
| Congo | 2011/12 | DHS | Yes | Yes | Yes | Yes | All | Yes | <http://measuredhs.com> | [14] |
| CAR | 2006 | MICS | Yes | No | Yes | Yes | One | No | <http://www.childinfo.org> | [15] |
| CAR | 2010 | MICS | Yes | Yes | Yes | Yes | All | Yes | <http://www.childinfo.org> | [16] |
| Comoros | 2012 | DHS | Yes | Yes | Yes | Yes | All | Yes | <http://measuredhs.com> | [17] |
| Cote d'Ivoire | 2005 | AIS | Yes | Yes | Yes | No | All | Yes | <http://measuredhs.com> | [18] |
| Cote d'Ivoire | 2006 | MICS | Yes | No | Yes | Yes | One | No | <http://www.childinfo.org> | [19] |
| Cote d'Ivoire | 2011/12 | DHS | Yes | Yes | Yes | Yes | All | Yes | <http://measuredhs.com> | [20] |
| DRC | 2007 | DHS | Yes | Yes | Yes | No | All | Yes | <http://measuredhs.com> | [21] |
| DRC | 2010 | MICS | Yes | Yes | Yes | Yes | All | Yes | <http://www.childinfo.org> | [22] |
| DRC | 2012/13 | DHS | Yes | Yes | Yes | Yes | All | Yes | <http://measuredhs.com> | [23] |
| Djibouti | 2006 | MICS | Yes | No | Yes | No | One | No | <http://www.childinfo.org> | [24] |
| Eritrea | 2008 | MIS | Yes | Yes | Yes | Yes | Yes | Yes | NMCP | [25] |
| Ethiopia | 2005 | DHS | Yes | Yes | Yes | Yes | All | Yes | <http://measuredhs.com> | [26] |
| Gabon | 2012 | DHS | Yes | Yes | Yes | Yes | All | Yes | <http://measuredhs.com> | [27] |
| Gambia | 2005/06 | MICS | Yes | No | Yes | Yes | One | No | <http://www.childinfo.org> | [28] |
| Ghana | 2003 | DHS | Yes | Yes | Yes | Yes | All | Yes | <http://measuredhs.com> | [29] |
| Ghana | 2006 | MICS | Yes | No | Yes | Yes | One | No | <http://www.childinfo.org> | [30] |
| Ghana | 2008 | DHS | Yes | Yes | Yes | Yes | All | Yes | <http://measuredhs.com> | [31] |
| Ghana | 2011 | MICS | Yes | Yes | Yes | Yes | All | Yes | <http://www.childinfo.org> | [32] |
| Guinea | 2005 | DHS | Yes | Yes | Yes | No | All | Yes | <http://measuredhs.com> | [33] |
| Guinea | 2012 | DHS | Yes | Yes | Yes | Yes | All | Yes | <http://measuredhs.com> | [34] |
| Guinea Bissau | 2006 | MICS | Yes | No | Yes | Yes | One | No | <http://www.childinfo.org> | [35] |
| Kenya | 2003 | DHS | Yes | Yes | Yes | No | All | Yes | <http://measuredhs.com> | [36] |
| Kenya | 2008/09 | DHS | Yes | Yes | Yes | Yes | All | Yes | <http://measuredhs.com> | [37] |
| Kenya | 2010 | MIS | Yes | Yes | Yes | Yes | All | Yes | <http://statistics.knbs.or.ke/nada> | [38] |
| Liberia | 2009 | MIS | Yes | Yes | Yes | Yes | All | Yes | <http://measuredhs.com> | [39] |
| Liberia | 2011 | MIS | Yes | Yes | Yes | Yes | All | Yes | <http://measuredhs.com> | [40] |
| Liberia | 2013 | DHS | Yes | Yes | Yes | Yes | All | Yes | <http://measuredhs.com> | [41] |
| Madagascar | 2008/09 | DHS | Yes | Yes | Yes | Yes | All | Yes | <http://measuredhs.com> | [42] |
| Madagascar | 2011 | MIS | Yes | Yes | Yes | Yes | All | Yes | <http://measuredhs.com> | [43] |
| Madagascar | 2013 | MIS | Yes | Yes | Yes | Yes | All | Yes | <http://measuredhs.com> | [44] |
| Malawi | 2004 | DHS | Yes | Yes | Yes | No | All | Yes | <http://measuredhs.com> | [45] |
| Malawi | 2006 | MICS | Yes | No | Yes | No | One | No | <http://www.childinfo.org> | [46] |
| Malawi | 2010 | DHS | Yes | Yes | Yes | Yes | All | Yes | <http://measuredhs.com> | [47] |
| Malawi | 2012 | MIS | Yes | Yes | Yes | Yes | All | Yes | <http://measuredhs.com> | [48] |
| Mali | 2006 | DHS | Yes | Yes | Yes | Yes | All | Yes | <http://measuredhs.com> | [49] |
| Mali | 2010 | EA&P | Yes | Yes | Yes | Yes | All | Yes | <http://measuredhs.com> | [50] |
| Mali | 2012/13 | DHS | Yes | Yes | Yes | Yes | All | Yes | <http://measuredhs.com> | [51] |
| Mauritania | 2007 | MICS | Yes | No | Yes | No | One | No | <http://www.childinfo.org> | [52] |
| Mozambique | 2011 | DHS | Yes | Yes | Yes | Yes | All | Yes | <http://measuredhs.com> | [53] |
| Namibia | 2006/07 | DHS | Yes | Yes | Yes | Yes | All | Yes | <http://measuredhs.com> | [54] |
| Namibia | 2009 | MIS | Yes | Yes | Yes | Yes | All | Yes | <http://www.mhss.gov.na> | [55] |
| Niger | 2006 | DHS | Yes | Yes | Yes | Yes | All | Yes | <http://measuredhs.com> | [56] |
| Niger | 2012 | DHS | Yes | Yes | Yes | Yes | All | Yes | <http://measuredhs.com> | [57] |
| Nigeria | 2003 | DHS | Yes | Yes | Yes | Yes | All | Yes | <http://measuredhs.com> | [58] |
| Nigeria | 2007 | MICS | Yes | No | Yes | Yes | One | No | <http://www.childinfo.org> | [59] |
| Nigeria | 2008 | DHS | Yes | Yes | Yes | Yes | All | Yes | <http://measuredhs.com> | [60] |
| Nigeria | 2010 | MIS | Yes | Yes | Yes | Yes | All | Yes | <http://measuredhs.com> | [61] |
| Nigeria | 2011 | MICS | Yes | Yes | Yes | Yes | All | Yes | <http://www.childinfo.org> | [62] |
| Nigeria | 2013 | DHS | Yes | Yes | Yes | Yes | All | Yes | <http://measuredhs.com> | [63] |
| Rwanda | 2005 | DHS | Yes | Yes | Yes | Yes | All | Yes | <http://measuredhs.com> | [64] |
| Rwanda | 2007/08 | DHS | Yes | Yes | Yes | Yes | All | Yes | <http://measuredhs.com> | [65] |
| Rwanda | 2010 | DHS | Yes | Yes | Yes | Yes | All | Yes | <http://measuredhs.com> | [66] |
| STP | 2008/09 | DHS | Yes | Yes | Yes | Yes | All | Yes | <http://measuredhs.com> | [67] |
| Senegal | 2005 | DHS | Yes | Yes | Yes | Yes | All | Yes | <http://measuredhs.com> | [68] |
| Senegal | 2006 | MIS | Yes | Yes | Yes | Yes | All | Yes | <http://measuredhs.com> | [69] |
| Senegal | 2008/09 | MIS | Yes | Yes | Yes | Yes | All | Yes | <http://measuredhs.com> | [70] |
| Senegal | 2010/11 | DHS | Yes | Yes | Yes | Yes | All | Yes | <http://measuredhs.com> | [71] |
| Senegal | 2012/13 | DHS | Yes | Yes | Yes | Yes | All | Yes | <http://measuredhs.com> | [72] |
| Sierra Leone | 2005 | MICS | Yes | No | Yes | Yes | One | No | <http://www.childinfo.org> | [73] |
| Sierra Leone | 2008 | DHS | Yes | Yes | Yes | Yes | All | Yes | <http://measuredhs.com> | [74] |
| Sierra Leone | 2010 | MICS | Yes | Yes | Yes | Yes | All | Yes | <http://www.childinfo.org> | [75] |
| Sierra Leone | 2013 | DHS | Yes | Yes | Yes | Yes | All | Yes | <http://measuredhs.com> | [76] |
| Somalia | 2006 | MICS | Yes | No | Yes | Yes | One | No | <http://www.childinfo.org> | [77] |
| Swaziland | 2006 | DHS | Yes | Yes | Yes | Yes | All | Yes | <http://measuredhs.com> | [78] |
| Swaziland | 2010 | MICS | Yes | Yes | Yes | Yes | All | Yes | <http://www.childinfo.org> | [79] |
| Tanzania | 2004/05 | DHS | Yes | Yes | Yes | No | All | Yes | <http://measuredhs.com> | [80] |
| Tanzania | 2007/08 | MIS | Yes | Yes | Yes | Yes | All | Yes | <http://measuredhs.com> | [81] |
| Tanzania | 2010 | DHS | Yes | Yes | Yes | Yes | All | Yes | <http://measuredhs.com> | [82] |
| Tanzania | 2011/12 | MIS | Yes | Yes | Yes | Yes | All | Yes | <http://measuredhs.com> | [83] |
| Togo | 2006 | MICS | Yes | No | Yes | Yes | One | No | <http://www.childinfo.org> | [84] |
| Togo | 2010 | MICS | Yes | Yes | Yes | Yes | All | Yes | <http://www.childinfo.org> | [85] |
| Uganda | 2006 | DHS | Yes | Yes | Yes | Yes | All | Yes | <http://measuredhs.com> | [86] |
| Uganda | 2009 | MIS | Yes | Yes | Yes | Yes | All | Yes | <http://measuredhs.com> | [87] |
| Uganda | 2011 | DHS | Yes | Yes | Yes | Yes | All | Yes | <http://measuredhs.com> | [88] |
| Zambia | 2001/02 | DHS | Yes | Yes | Yes | No | All | Yes | <http://measuredhs.com> | [89] |
| Zambia | 2007 | DHS | Yes | Yes | Yes | Yes | All | Yes | <http://measuredhs.com> | [90] |
| Zambia | 2010 | MIS | No | Yes | No | No | N/A | Yes | NMCP | [91] |
| Zambia | 2012 | MIS | No | Yes | No | No | N/A | Yes | NMCP | [92] |
| Zimbabwe | 2005/06 | DHS | Yes | Yes | Yes | Yes | All | Yes | <http://measuredhs.com> | [93] |
| Zimbabwe | 2009 | MICS | Yes | No | Yes | Yes | One | No | <http://www.childinfo.org> | [94] |
| Zimbabwe | 2010/11 | DHS | Yes | Yes | Yes | Yes | All | Yes | <http://measuredhs.com> | [95] |
